# Band Offset Measurements in Atomic-Layer-Deposited Al_2_O_3_/Zn_0.8_Al_0.2_O Heterojunction Studied by X-ray Photoelectron Spectroscopy

**DOI:** 10.1186/s11671-017-2131-8

**Published:** 2017-05-19

**Authors:** Baojun Yan, Shulin Liu, Yuekun Heng, Yuzhen Yang, Yang Yu, Kaile Wen

**Affiliations:** 1 State Key Laboratory of Particle Detection and Electronics, Institute of High Energy Physics of Chinese Academy of Sciences, Beijing, 100049 People’s Republic of China; 2 Department of Physics, Nanjing University, Nanjing, 210093 People’s Republic of China; 3 School of Science, Xi’an University of Technology, Xi’an, 710048 People’s Republic of China; 4 University of Chinese Academy of Sciences, Beijing, 100049 People’s Republic of China

**Keywords:** Atomic layer deposition, X-ray photoelectron spectroscopy, Heterojunction, Microchannel plate

## Abstract

Pure aluminum oxide (Al_2_O_3_) and zinc aluminum oxide (Zn_*x*_Al_1-*x*_O) thin films were deposited by atomic layer deposition (ALD). The microstructure and optical band gaps (*E*_*g*_) of the Zn_*x*_Al_1-*x*_O (0.2 ≤ *x* ≤ 1) films were studied by X-ray diffractometer and Tauc method. The band offsets and alignment of atomic-layer-deposited Al_2_O_3_/Zn_0.8_Al_0.2_O heterojunction were investigated in detail using charge-corrected X-ray photoelectron spectroscopy. In this work, different methodologies were adopted to recover the actual position of the core levels in insulator materials which were easily affected by differential charging phenomena. Valence band offset (Δ*E*_V_) and conduction band offset (Δ*E*_C_) for the interface of the Al_2_O_3_/Zn_0.8_Al_0.2_O heterojunction have been constructed. An accurate value of Δ*E*_V_ = 0.82 ± 0.12 eV was obtained from various combinations of core levels of heterojunction with varied Al_2_O_3_ thickness. Given the experimental *E*_*g*_ of 6.8 eV for Al_2_O_3_ and 5.29 eV for Zn_0.8_Al_0.2_O, a type-I heterojunction with a Δ*E*_C_ of 0.69 ± 0.12 eV was found. The precise determination of the band alignment of Al_2_O_3_/Zn_0.8_Al_0.2_O heterojunction is of particular importance for gaining insight to the design of various electronic devices based on such heterointerface.

## Background

Nano-thick oxide films with high resistance have attracted much attention as the most promising conductive layer for the applications of microchannel plate (MCP) as electron multiplier [1, 2], resistive memories [3], and electron-optical micro-electro mechanical systems (MEMS) [4]. A large research effort has been devoted to the novel idea of adjusting resistivity of such thin films due to the abovementioned large potential applications in a special environment. MCP is a thin glass plate with thickness of about 500 μm consisting of several millions pores of a cylinder geometry with a 4–25-μm diameter and with a bias angle usually 5°–13° to the normal of the plate surface, and the high aspect ratio in each pore is about 20:1–100:1 [5, 6]. For recent MCP fabrication, two kinds of nano-thick layers are deposited on the MCP pore surfaces to conduct an electron multiplication function [1, 2]. The first layer is a conductive layer for supplying electrons, and the second layer is a secondary electron emission (SEE) layer for generating electrons. The three-dimensional surfaces and high aspect ratio of MCP should be firstly taken into consideration for depositing uniform thickness and composition of thin films. So far, the only effective approach growing high-quality thin films is the atomic layer deposition (ALD) technique based on sequential self-terminating gas-solid reactions [7].

ZnO is an n-type semiconductor with a direct bandgap of around 3.37 eV and a large exciton binding energy of 60 meV at room temperature [8, 9]. A lot of elements such as Mg [10, 11], Cd [12], Ga [13], W [14], and Mo [15] were used to doping in ZnO in order to tune its optical and electrical properties for special applications. In electron multiplier application, such as MCP, zinc aluminum oxide (Zn_*x*_Al_1-*x*_O) films have been investigated because of their thermal stability in a special application environment and low cost of industrialization. The properties of Zn_*x*_Al_1-*x*_O films can be controlled by changing the Al content, paving a way to design optoelectronic and photonic devices based on this material. Usually, high-resistivity Zn_*x*_Al_1-*x*_O thin films as a conductive layer with *x* at the range of 0.7–0.85 have been applied in the field of electron multiplier [16]. For SEE layers, boron-doped diamond with hydrogen-terminated material has higher SEE coefficient than that of other traditional insulators. This provides a strong impetus for the development of electron multipliers. However, in the presence of degradation due to electron beam-induced contamination, these must be seriously regarded as preliminary [17]. From a practical point of view, two kinds of traditional insulators used as SEE layers in MCP are magnesium oxide (MgO) and Al_2_O_3_ thin films [18]. Although pure MgO has higher SEE coefficient than that of Al_2_O_3_, it is limited in the application on MCP because it is highly deliquescent and its surface is rather reactive with atmospheric moisture and carbon dioxide as demonstrated by our previous work [19], which probably results in degraded SEE performance. However, the physical and chemical properties of Al_2_O_3_ are very stable even after long-term exposure to the atmosphere. Therefore, Al_2_O_3_ is one of the most commonly used SEE materials in MCP application.

According to the structure of MCP, the Al_2_O_3_ and Zn_*x*_Al_1-*x*_O thin films have different band gaps (*E*_g_) resulting in band offsets in the heterointerface. Therefore, the determination of the band offsets at Al_2_O_3_/Zn_*x*_Al_1-*x*_O interface is of importance because valence band offset (Δ*E*_V_) and conduction band offset (Δ*E*_C_) can deteriorate or promote SEE performance and also have a great influence on the performance of electron multiplier.

Generally, Kraut’s method is widely used to calculate the valence band maximum (VBM) and the conduction band minimum (CBM) of semiconductor/semiconductor heterojunctions [20]. However, in the case of insulator/semiconductor or, in more serious cases, insulator/insulator heterojunctions, the positive charges generated during X-ray bombardment accumulate in the insulators and induce a strong modification of the kinetic energy of the emitted photoelectrons which is the so-called differential charging effect [21]. Although it is probably dealt with using a neutralizing electron gun [22], the use of C 1*s* peak recalibration [23], and zero charging method [24–26], a careful evaluation of the experimental result is necessary due to the differential charging effect during X-ray irradiation [19].

In this work, we will study the structure and optical *E*_g_ of Zn_*x*_Al_1-*x*_O (0.2 ≤ *x* ≤ 1) thin films firstly, and then, we especially determine the Δ*E*_V_ and Δ*E*_C_ of the Al_2_O_3_/Zn_0.8_Al_0.2_O heterojunction by using high-resolution X-ray photoelectron spectroscopy (XPS).

## Methods

### Sample Preparation

Several samples were used in this study: nine 80-nm-thick Zn_*x*_Al_1-*x*_O samples (0.2 ≤ *x* ≤ 1) individually grown on n-Si (1 1 1) and quartz substrates, a 30-nm-thick Al_2_O_3_ grown on n-Si (1 1 1) substrate, and 3, 4, 5, 8 nm of Al_2_O_3_ on 80 nm of Zn_0.8_Al_0.2_O grown on n-Si (1 1 1). The quartz substrates were ultrasonically cleaned in an ethanol/acetone solution and then rinsed in deionized water. The polished Si substrates were dipped in hydrofluoric acid for 30 s and then placed in an ALD chamber waiting for deposition. For Zn_*x*_Al_1-*x*_O layer deposition, ZnO:Al_2_O_3_ ALD was carried out using diethylzinc (DEZ), trimethylaluminum (TMA), and deionized water as Zn, Al, and oxidant precursor, respectively. The Al_2_O_3_ ALD was performed using separate TMA and H_2_O exposures with sequence TMA/N_2_/H_2_O/N_2_ (150 ms/4 s/150 ms/4 s). The ZnO ALD was performed using separate DEZ and H_2_O exposures following the sequence DEZ/N_2_/H_2_O/N_2_ (150 ms/4 s/150 ms/4 s). The doping was carried out by substituting TMA exposure for DEZ. The Zn contents in the Zn_*x*_Al_1-*x*_O layers were controlled by adjusting the ratio of the pulse cycles of DEZ and TMA, where the Zn content *x* was varied from 0.2 to 1 (pure ZnO) atom %. For Zn_0.8_Al_0.2_O layer, the DEZ and H_2_O pulses were alternated, and every fifth DEZ pulse was substituted with a TMA pulse. Ultrahigh purity nitrogen was used as a carrier and purge gas. The reaction temperatures were 200 °C. The detailed parameters are listed in Table 1.

**Table 1:** Detailed parameters for Zn_0.8_Al_0.2_O and Al_2_O_3_ layers

Sample no.	Substrate	Material	Thickness (nm)	Composition	Characterization
ALD01	n-Si (111), quartz	Zn_0.8_Al_0.2_O	80	Zn:Al = 4:1 (atomic ratio)	XPS, XRD and UV-Vis
ALD02	n-Si (111)	Al_2_O_3_	30	Pure	XPS
ALD03	n-Si/ALD01	Al_2_O_3_	3	Pure	XPS, SE
ALD04	n-Si/ALD01	Al_2_O_3_	4	Pure	XPS, SE
ALD05	n-Si/ALD01	Al_2_O_3_	5	Pure	XPS, SE
ALD06	n-Si/ALD01	Al_2_O_3_	8	Pure	XPS, SE

### Characterization

Optical transmittance spectra in a wavelength range from 185 to 700 nm were carried out by using a double-beam UV-Vis-IR spectrophotometer (Agilent Cary 5000) at room temperature in air. The crystal structure of the films were characterized by X-ray diffraction (XRD, Bruker D8) using Cu *K*_*α*_ radiation (40 kV, 40 mA, *λ* = 1.54056 Å). The film thickness was measured by Spectroscopic Ellipsometry (Sopra GES5E) where the incident angle was fixed at 75°, and the wavelength region from 230 to 900 nm was scanned with 5-nm steps. And the ellipsometric thicknesses of samples ALD03, ALD04, ALD05, and ALD06 were 3.01, 4.02, 5.01, and 8.01 nm, respectively. The XPS (PHI Quantera SXM) is used to analyze both the core levels (CLs) and valence band spectra of the samples. Charge neutralization was performed with an electron flood gun, and all XPS spectra were calibrated by the C 1*s* peak at 284.6 eV. In order to avoid differential charging effect, during the measurement, the spectra were taken after a few minutes of X-ray irradiation. All the samples are measured under the same conditions in order to acquire reliable data.

### Calculations

The Δ*E*_V_ of the Al_2_O_3_/Zn_0.8_Al_0.2_O heterojunction can be calculated from Kraut’s formula

1 $$ \varDelta {E}_{\mathrm{V}}=\left({E}_{\mathrm{CL}}^{{\mathrm{Zn}}_{0.8}{\mathrm{Al}}_{0.2}\mathrm{O}}(y)-{E}_{\mathrm{V}\mathrm{BM}}^{{\mathrm{Zn}}_{0.8}{\mathrm{Al}}_{0.2}\mathrm{O}}\right)\hbox{-} \left({E}_{\mathrm{CL}}^{{\mathrm{Al}}_2{\mathrm{O}}_3}(x)-{E}_{\mathrm{V}\mathrm{BM}}^{{\mathrm{Al}}_2{\mathrm{O}}_3}\right)\hbox{-} \varDelta {E}_{\mathrm{CL}} $$

where $\varDelta {E}_{\mathrm{CL}}=\left({E}_{\mathrm{CL}}^{{\mathrm{Zn}}_{0.8}{\mathrm{Al}}_{0.2}\mathrm{O}}(y)-{E}_{\mathrm{CL}}^{{\mathrm{Al}}_2{\mathrm{O}}_3}(x)\right)$ was the energy difference between feature *y* and feature *x* CLs, which were measured by XPS measurement in the heterojunction sample, and $\left({E}_{\mathrm{CL}}^{{\mathrm{Al}}_2{\mathrm{O}}_3}(x)-{E}_{\mathrm{VBM}}^{{\mathrm{Al}}_2{\mathrm{O}}_3}\right)$ and $\left({E}_{\mathrm{CL}}^{{\mathrm{Zn}}_{0.8}{\mathrm{Al}}_{0.2}\mathrm{O}}(y)-{E}_{\mathrm{VBM}}^{{\mathrm{Zn}}_{0.8}{\mathrm{Al}}_{0.2}\mathrm{O}}\right)$ were the Al_2_O_3_ and Zn_0.8_Al_0.2_O bulk constants, which were obtained on the respective thick films. The VBM values were determined by linear extrapolation of the leading edge to the baseline of the valence band spectra. A root sum square relationship is used to combine the uncertainties in the different binding energies to determine the uncertainty of calculated results [26].

## Results and Discussion

### Structure and Band Gaps of Zn_*x*_Al_1-*x*_O Samples

**Fig. 1:**
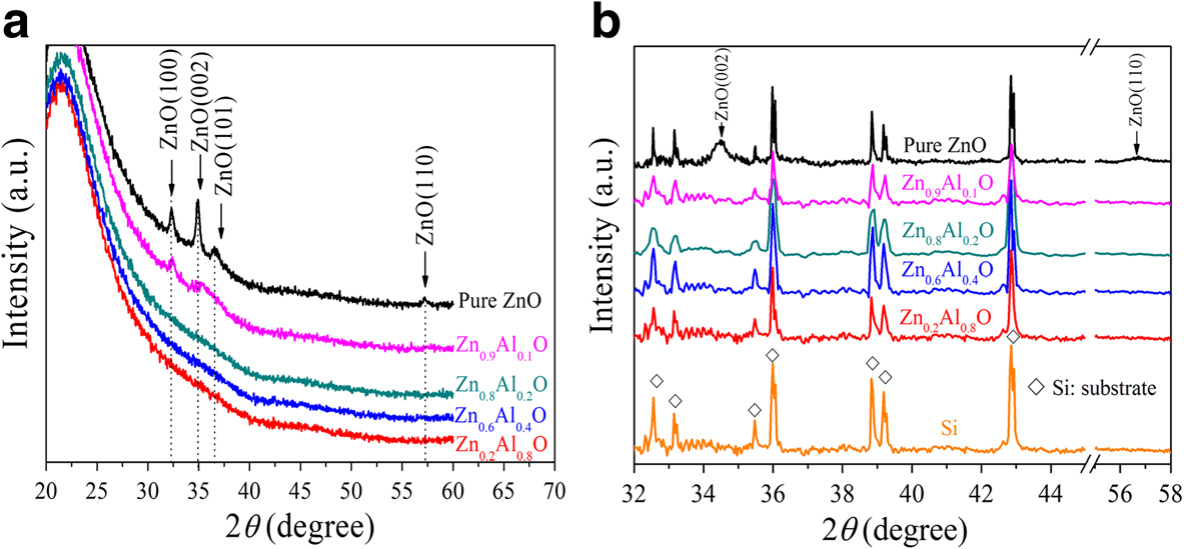
XRD patterns of Zn_*x*_Al_1-*x*_O samples deposited on **a** quartz substrate and **b** Si substrate

The XRD patterns of the as-deposited 80-nm-thick Zn_*x*_Al_1-*x*_O (*x* = 0.2, 0.6, 0.8, 0.9, 1) thin films grown on quartz and Si substrates are shown in Fig. 1a, b, respectively. For the pure ZnO grown on quartz substrates in Fig. 1a, the strong peaks at 32.4° and 34.8° and the relatively weak peaks at 36.5° and 57.2° come from hexagonal ZnO phase, indicating the polycrystalline nature of the ZnO layer. And strong (0 0 2) peak shows the preferential orientation growth of ALD ZnO. However, the above characteristic peaks become weak for Zn_0.9_Al_0.1_O sample and disappear for Zn_*x*_Al_1-*x*_O (*x* ≤ 0.8) samples, which suggests that ZnO crystallization is suppressed with Al concentration increase. Besides, the broad peak ranging from 20° to 30° is the typical pattern of the quartz substrate. For Si substrate, the strong peaks around 28.4° and 58.9° are easily detected (data not shown). These peaks are corresponding to the diffractions originated from Si (1 1 1) and Si (2 2 2) crystal planes. In addition, the relatively weak peaks in Fig. 1b at 2*θ* = 32.6°, 33.2°, 35.4°, 35.9°, 38.8°, 39.2°, and 42.8° in the diffractograms that arise from the Si substrate itself are also observed. These unknown peaks may be related to the process conditions for producing crystalline silicon and are observed in previous work [27, 28]. Except for diffraction peaks from the Si substrate, no other diffraction peaks from the Zn_*x*_Al_1-*x*_O (*x* ≤ 0.9) samples are detected. Only (0 0 2) and weak (1 1 0) peaks appear in the pure ZnO sample. From the above results, the crystal quality of the Zn_*x*_Al_1-*x*_O film is a serious decline with the increasing concentration of Al content. It is well known that the particle size of Al ions is less than that of Zn ions. Zn is easily substituted by Al when doping concentration of Al increases. This results in weakened ZnO crystallinity, so the structure of Zn_*x*_Al_1-*x*_O (*x* ≤ 0.8) samples is amorphous, in good agreement with previous results [29]. Taken into consideration, Zn_*x*_Al_1-*x*_O layer growth appears to be substrate sensitive and Al doping concentration has an influence on the crystallization of the films.

**Fig. 2:**
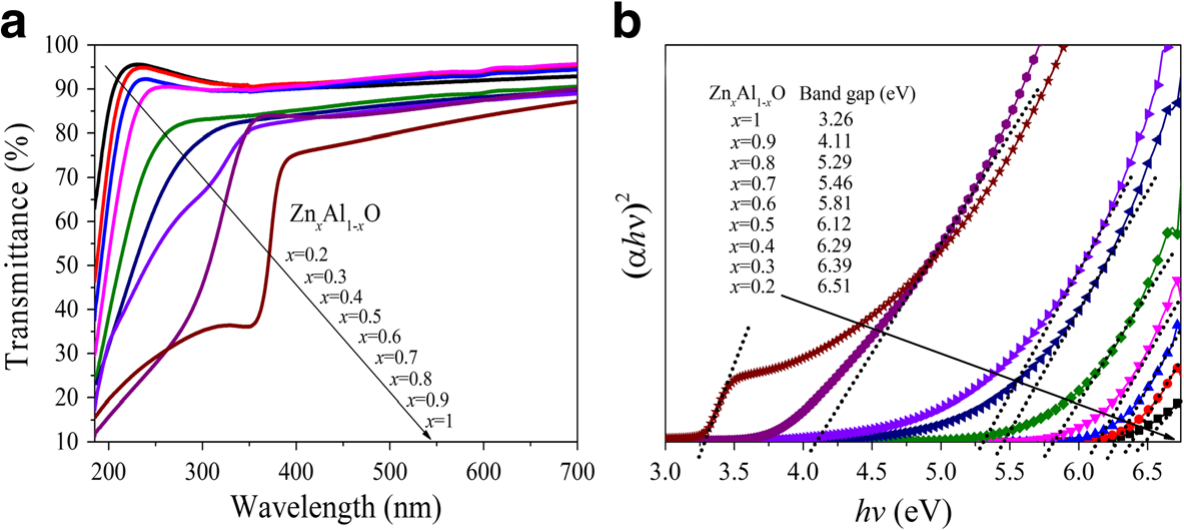
Transmittance spectra (**a**) and the plots of (*αhν*)^2^ vs. photon energy (**b**) of Zn_*x*_Al_1-*x*_O samples

Figure 2a shows transmission spectra of the Zn_*x*_Al_1-*x*_O samples deposited on quartz substrate. The average transmittance is above 80% in the visible wavelength for all samples. It is found that ZnO film exhibits abrupt absorption edge which appears at ~390 nm corresponding to the fundamental *E*_g_ of ZnO. A blue shift of the absorption edge is apparently observed when Al concentration increases. The *E*_g_ of Zn_*x*_Al_1-*x*_O thin films can be obtained by fitting the sharp absorption edges. The relationship between absorption coefficient (*α*) and *E*_g_ of direct band gap semiconductor is given by Tauc equation [30], (*αhv*)^2^ = B(*hv*−*E*_g_), where *hν* is the photon energy and B is a constant. The dependence of (*αhν*)^2^ on photon energy is shown in Fig. 2b. The *E*_*g*_ is obtained by the extrapolations of the liner regions of the optical absorption edges. The *E*_g_ of pure ZnO thin film deposited by ALD is 3.26 eV, which is consistent with the previous reports [31, 32]. With the Zn concentration *x* decreases from 0.9 to 0.2, the *E*_g_ of Zn_*x*_Al_1-*x*_O thin films increases from 4.11 to 6.51 eV. It is directly demonstrated that the *E*_g_ of Zn_*x*_Al_1-*x*_O thin films can be adjusted in a large range by controlling the Al doping concentration, which makes it a suitable candidate for application in many scientific research fields [33, 34]. For the new type of MCP, the properties of Zn_0.8_Al_0.2_O thin film are suitable for conductive layer proved by previous study [2]. Therefore, the *E*_g_ of atomic-layer-deposited Zn_0.8_Al_0.2_O thin film is 5.29 eV, which is sufficient to make a band gap discontinuity in Al_2_O_3_/Zn_0.8_Al_0.2_O heterojunction and is used for calculating the Δ*E*_C_ value later.

### Valence and Conduction Band Offset Measurements of Al_2_O_3_/Zn_0.8_Al_0.2_O Heterojunction

**Fig. 3:**
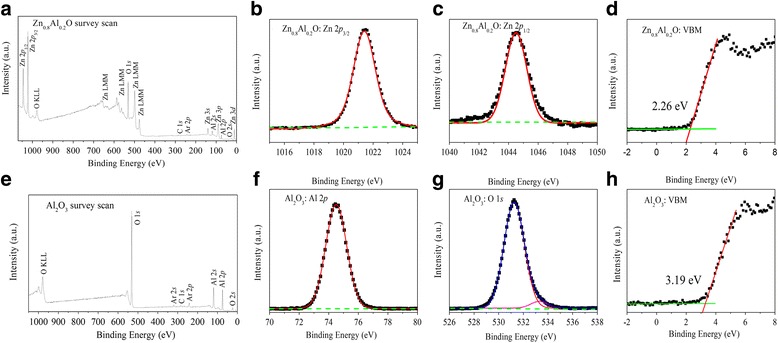
XPS spectra for **a** survey scan, **b** Zn 2*p*_3/2_, **c** Zn 2*p*_1/2_, and **d** VBM of Zn_0.8_Al_0.2_O and **e** survey scan, **f** Al 2*p*, **g** O 1*s*, and **h** VBM of Al_2_O_3_, with application of a low-energy electron flood gun

The XPS spectra of survey scan, CLs, and VBM region for Zn_0.8_Al_0.2_O and Al_2_O_3_ samples are shown in Fig. 3. In this study, we find that the CLs positions of the Zn_0.8_Al_0.2_O and Al_2_O_3_ thin films do not change as a function of X-ray irradiation time for 15 min (data not shown), because of operating a low energy electron flood gun. Figure 3a, e shows the whole scanning spectrum of the thick Zn_0.8_Al_0.2_O and Al_2_O_3_ thin films, respectively. The C 1*s* peak at 284.6 eV appeared due to some surface contamination, and the Ar 2*p* peak at 242.1 eV appeared because of residual inert gas composition in the ultrahigh vacuum chamber. The peaks in Fig. 3a located 660, 652, 582, 573, 559, 495, and 472 eV are Auger lines of Zn element. The stoichiometry of the thick films are checked by the ratio of the integrated area of Zn 2*p* peak to Al 2*p* peak for the Zn_0.8_Al_0.2_O sample and Al 2*p* peak to O 1 *s* peak for the Al_2_O_3_ sample. Both are corrected by corresponding atomic sensitivity factors *S* [35], taking into account their corresponding photoionization cross-sections of CLs calculated by Scofield [36], and the mean free path of the photoelectrons calculated by Tanuma et al [37]. Here, the *S* values are calculated to be 0.256, 2.768, and 0.733 for Al 2*p*, Zn 2*p*_3/2_, and O 1*s*. The atomic ratios Zn:Al = 3.97:1.01 for Zn_0.8_Al_0.2_O and Al:O = 1.99:3.01 for Al_2_O_3_ compare well with that of designed ratio of atomic layer deposition, which indicate good stoichiometry of the Zn_0.8_Al_0.2_O and Al_2_O_3_ layers. The high-resolution scans of Zn 2*p*_3/2_ and Zn 2*p*_1/2_ CLs of Zn_0.8_Al_0.2_O are shown in Fig. 3b, c. The peaks fitted using Shirley backgrounds and Voigt (mixed Lorentzian-Gaussian) functions located 1021.41 and 1044.51 eV in Fig. 3b, c correspond to the electronic states of Zn 2*p*_3/2_ and Zn 2*p*_1/2_, respectively, and both are fitted by a single contribution, attributed to the bonding configuration Zn-O. The Al 2*p* peak of Al_2_O_3_ located 74.35 eV and O 1*s* peak located 531.1 eV are shown in Fig. 3f, g. The Al 2*p* spectrum as fitted by a single contribution, attributed to the bonding configuration Al-O. However, for the O 1*s* spectrum, an additional peak low-intensity higher binding energy component is also observed. This extra component is attributed to both O-Al and O-H bonds [38]. The VBM positions are determined by a linear extrapolation of the leading edge of the valence band spectrum and the background [39], as shown in Fig. 3d,h. This linear method has already been widely used to determine the VBM of semiconductors with high accuracy. The VBM values of atomic-layer-deposited thick Zn_0.8_Al_0.2_O and Al_2_O_3_ samples are 2.26 and 3.19 eV, respectively. The scatter of the data relative to the fit are estimated as an uncertainty in VBM positions of less than 0.03 eV. The parameters deduced from Fig. 3 are summarized in Table 2 for clarity.

**Table 2:** Peak positions of CLs and VBM positions used to calculate the Δ*E*_V_ of the Al_2_O_3_/Zn_0.8_Al_0.2_O heterojunction

	Zn_0.8_Al_0.2_O	Al_2_O_3_	3 nm Al_2_O_3_/Zn_0.8_Al_0.2_O	4 nm Al_2_O_3_/Zn_0.8_Al_0.2_O	5 nm Al_2_O_3_/Zn_0.8_Al_0.2_O
Al 2*p*		74.35	74.6	74.93	74.87
O 1*s*		531.1	531.32	531.66	531.63
Zn 2*p*_3/2_	1021.41		1021.69	1022.05	1021.97
Zn 2*p*_1/2_	1044.51		1044.85	*1045.11*	**1045.64**
VBM	2.26	3.19			

The peak position of Zn 2*p*_1/2_ in 5-nm Al_2_O_3_ sample listed by a bold number

**Fig. 4:**
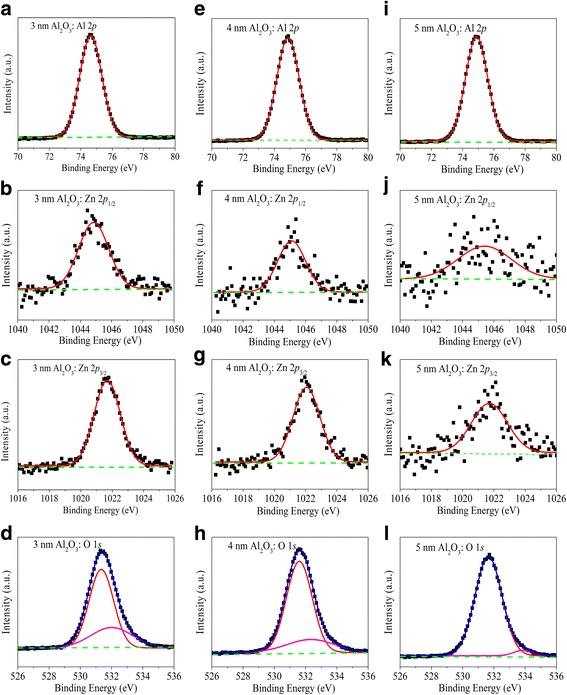
CLs of Al_2_O_3_/Zn_0.8_Al_0.2_O samples with varied Al_2_O_3_ thickness **a**–**d** 3 nm, **e**–**h** 4 nm, and **i**–**l** 5 nm, with application of a low-energy electron flood gun

Four CLs of Al_2_O_3_/Zn_0.8_Al_0.2_O heterojunction with different Al_2_O_3_ thickness are shown in Fig. 4. The Al 2*p*, Zn 2*p*_1/2_, and Zn 2*p*_3/2_ XPS spectra in Fig. 4(a, e, i), (b, f, j), and (c, g, k), respectively, are fitted by a single contribution, attributed to the bonding configurations Al-O and Zn-O. For the O 1*s* XPS spectrum in Fig. 4d, h, l, an additional low-intensity higher binding energy component is observed. The extra component is attributed to metal (Al, Zn)-O bonding at the interface and/or inelastic losses to free carries in the Al_2_O_3_ layer, similar results obtained by previous study [19]. With the increase of the Al_2_O_3_ thickness, the intensity of Zn 2*p*_1/2_ peak is weakened while the energy resolution is deteriorated shown in Fig. 4f. It is difficult to observe and fit for Al_2_O_3_ thickness of 5 nm as shown in Fig. 4j. So, the peak position of Zn 2*p*_1/2_ in 5-nm Al_2_O_3_ sample listed by a bold number in Table 2 is a large deviation as a result of the big error of fitting. The CLs of Al_2_O_3_/Zn_0.8_Al_0.2_O samples are summarized in Table 2.

The Δ*E*_V_ of the Al_2_O_3_/Zn_0.8_Al_0.2_O heterojunction is determined from the energy separation between the CLs in the Al_2_O_3_/Zn_0.8_Al_0.2_O sample and the VBM to CLs separations in the thick Al_2_O_3_ and Zn_0.8_Al_0.2_O samples, respectively. Table 3 lists the Δ*E*_V_ values for all Al_2_O_3_ samples with thickness of 3–5 nm, and the error in each value is ± 0.07 eV. Therefore, the averaged Δ*E*_V_ value is 0.87 ± 0.22 eV. It is important to note that the calculation does not include the italic numbers in Table 3 because of the big error fitting of CLs of Zn 2*p*_1/2_ in the 5-nm Al_2_O_3_/Zn_0.8_Al_0.2_O sample.

**Table 3:** The Δ*E*_V_ values of the Al_2_O_3_/Zn_0.8_Al_0.2_O heterojunction with Al_2_O_3_ thickness of 3–5 nm

Δ*E*_V_	3 nm Al_2_O_3_/Zn_0.8_Al_0.2_O		4 nm Al_2_O_3_/Zn_0.8_Al_0.2_O		5 nm Al_2_O_3_/Zn_0.8_Al_0.2_O	
	Zn 2*p*_3/2_	Zn 2*p*_1/2_	Zn 2*p*_3/2_	Zn 2*p*_1/2_	Zn 2*p*_3/2_	Zn 2*p*_1/2_
Al 2*p*	0.9	0.84	0.87	0.91	0.89	*0.32*
O 1 *s*	0.87	0.81	0.85	0.89	0.9	*0.33*

The calculation does not include the italic numbers

However, there are obvious considerable CL shifts up to 0.6 eV sensitive to the thicknesses of the Al_2_O_3_ and Zn_0.8_Al_0.2_O layers from the given experimental data in Table 2, and different Δ*E*_V_ values are obtained in the various combinations of XPS CLs in Table 3. It is directly proved that the charging phenomenon generated by the X-ray irradiation results in adverse effects on the Δ*E*_V_ determination when taking XPS measurement on insulator/semiconductor heterojunction in spite of operating neutralizing electron gun. As has been widely reported, the influences of differential charging on the band offsets determination cannot be neglected even in very thin oxides. Therefore, zero charging method is adopted in this study in order to eliminate charging-induced errors and recover the accurate Δ*E*_V_ value.

**Fig. 5:**
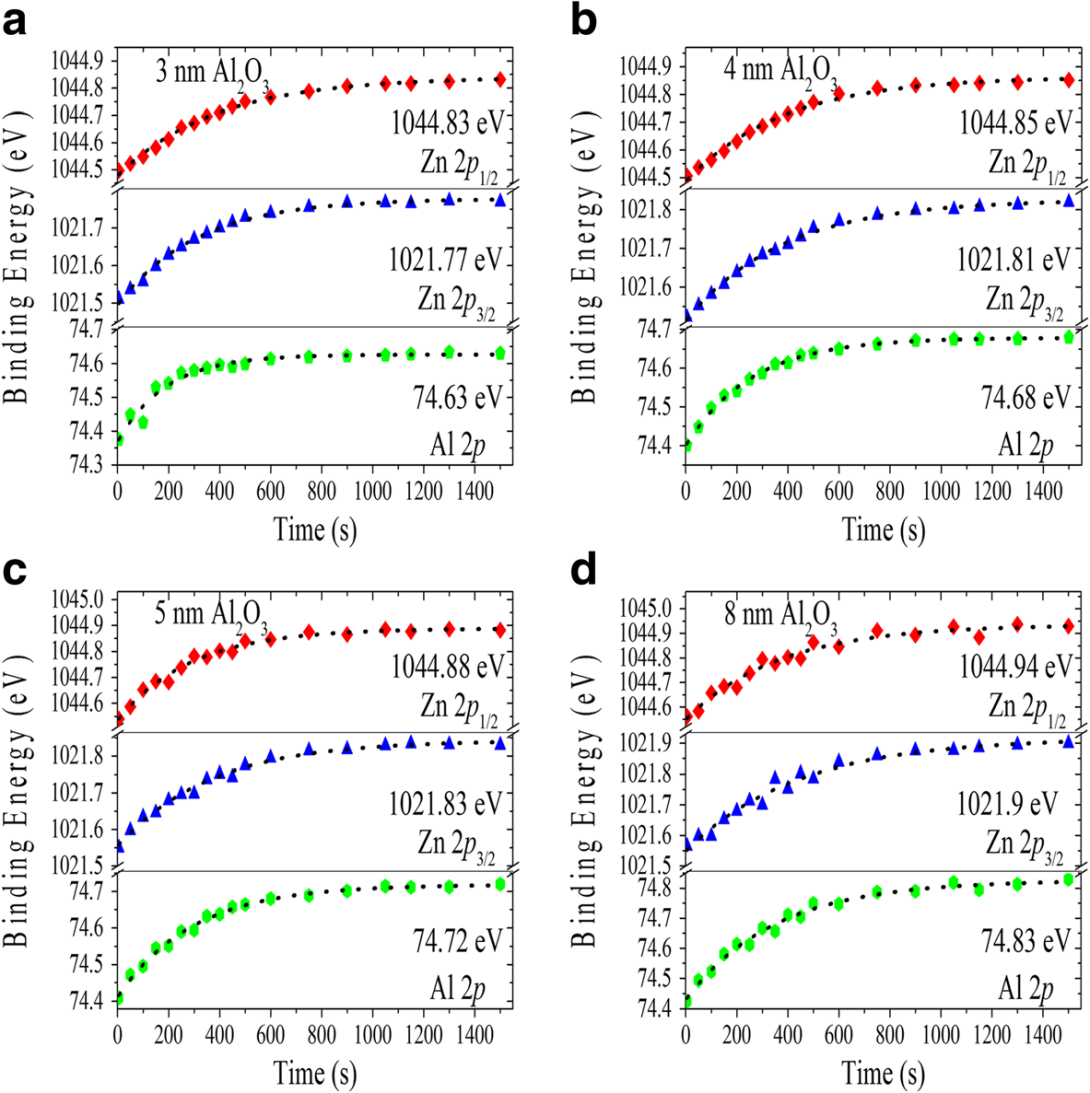
Time-resolved plots showing the respective binding energies vs. X-ray irradiation time for **a** 3 nm, **b** 4 nm, **c** 5 nm, and **d** 8 nm Al_2_O_3_ films on Zn_0.8_Al_0.2_O on Si substrates, with application of a low-energy electron flood gun

The error in the Δ*E*_V_ measurement is resulting from the differential charging effect that prevents the correct determination of the energy differences, such as between the Al 2*p* and Zn 2*p*_3/2_ signals even in very thin Al_2_O_3_ films in heterojunction. In Fig. 5, the binding energies of the Al 2*p*, Zn 2*p*_3/2_, and Zn 2*p*_1/2_ CLs for the 3, 4, 5, and 8 nm Al_2_O_3_ films are plotted as a function of X-ray irradiation time. The binding energies of Al 2*p*, Zn 2*p*_3/2_, and Zn 2*p*_1/2_ CLs of the 3-nm Al_2_O_3_ sample in Fig. 5a increase slowly until they stabilize on a steady state value of 74.63 ± 0.01, 1021.77 ± 0.01, and 1044.83 ± 0.02 eV, respectively. Similar time dependencies are observed in the 4-, 5-, and 8-nm Al_2_O_3_ films, as shown in Fig. 5b–d. The results show that CL steady state spectra are obtained after stabilization of the signals in the heterojunction-considered charge saturated when X-ray irradiation time is more than 25 min. Therefore, X-ray irradiation time is one of the most important parameters to determine the insulator/semiconductor band offsets. Layer thickness dependence in peak positions is mainly resulting from the differential charging effects. True peak positions can be acquired by extrapolating the measured binding energies to zero oxide thickness and ideally to zero charge, similar results are reported for SiO_2_/Si [25], HfO_2_/Si [26], and MgO/Zn_0.8_Al_0.2_O [19] systems.

**Fig. 6:**
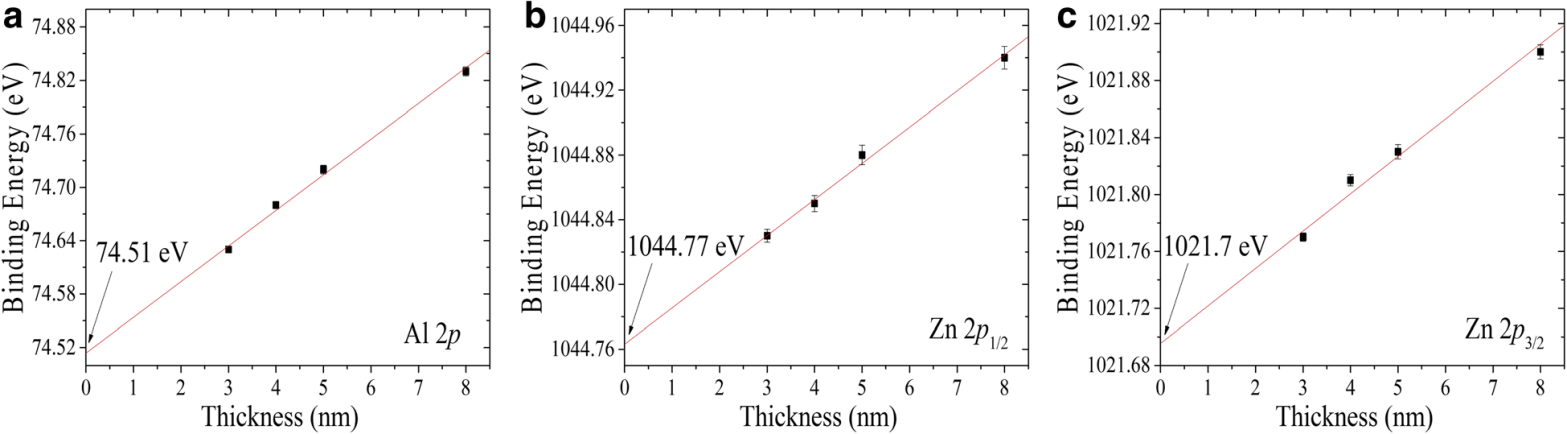
Al 2*p* (**a**), Zn 2*p*_1/2_ (**b**), and Zn 2*p*_3/2_ (**c**), CL binding energies as a function of the Al_2_O_3_ thin film thickness

The CLs positions of the Al 2*p*, Zn 2*p*_1/2_, and Zn 2*p*_3/2_ are plotted as a function of the Al_2_O_3_ film thickness, as shown in Fig. 6. By linear fitting of the experimental data, the CLs positions of the Al 2*p*, Zn 2*p*_1/2_, and Zn 2*p*_3/2_ peaks are determined to be 74.51 ± 0.03, 1044.77 ± 0.06, and 1021.7 ± 0.04 eV, respectively. In order to correct the Δ*E*_V_ of the Al_2_O_3_/Zn_0.8_Al_0.2_O heterojunction, we calculate the energy differences between the extrapolated (Al 2*p*, Zn 2*p*_1/2_) and (Al 2*p*, Zn 2*p*_3/2_) at zero thickness. The values are 970.26 ± 0.07 and 947.19 ± 0.05 eV, respectively. Inserting these values in Eq. (1), the Δ*E*_V_ are calculated to be 0.83 ± 0.09 and 0.8 ± 0.08 eV, which is in good agreement using the two combinations of CLs of the Al_2_O_3_/Zn_0.8_Al_0.2_O heterojunction. Therefore, the averaged Δ*E*_V_ value is 0.82 ± 0.12 eV.

There are three possible reasons that affect the Δ*E*_V_ values in addition to the XPS method itself. Firstly, the oxide stoichiometry of the Al_2_O_3_ thin films measured by XPS is almost the same in the different Al_2_O_3_ samples with thickness of 3–8 nm. Therefore, the composition of the Al_2_O_3_ film is independent of thickness and the binding energy shifts in Fig. 5 is related to the differential charging effect occurring in the Al_2_O_3_/Zn_0.8_Al_0.2_O heterojunction during X-ray irradiation. Secondly, band bending at the heterointerface could induce a systematic error in determination of Δ*E*_V_, and we check that this error is much smaller than the average standard deviation of ± 0.03 eV given above. Finally, the strain existing in the Al_2_O_3_ overlayer of the heterojunction will induce a piezoelectric field that probably affects the measured Δ*E*_V_ value, a similar phenomenon explained by Martin et al [40]. The heterojunction underlayer Zn_0.8_Al_0.2_O is thick enough, and the structure of both materials is amorphous. Therefore, the strain-induced piezoelectric field is not taken into consideration in this study.

**Fig. 7:**
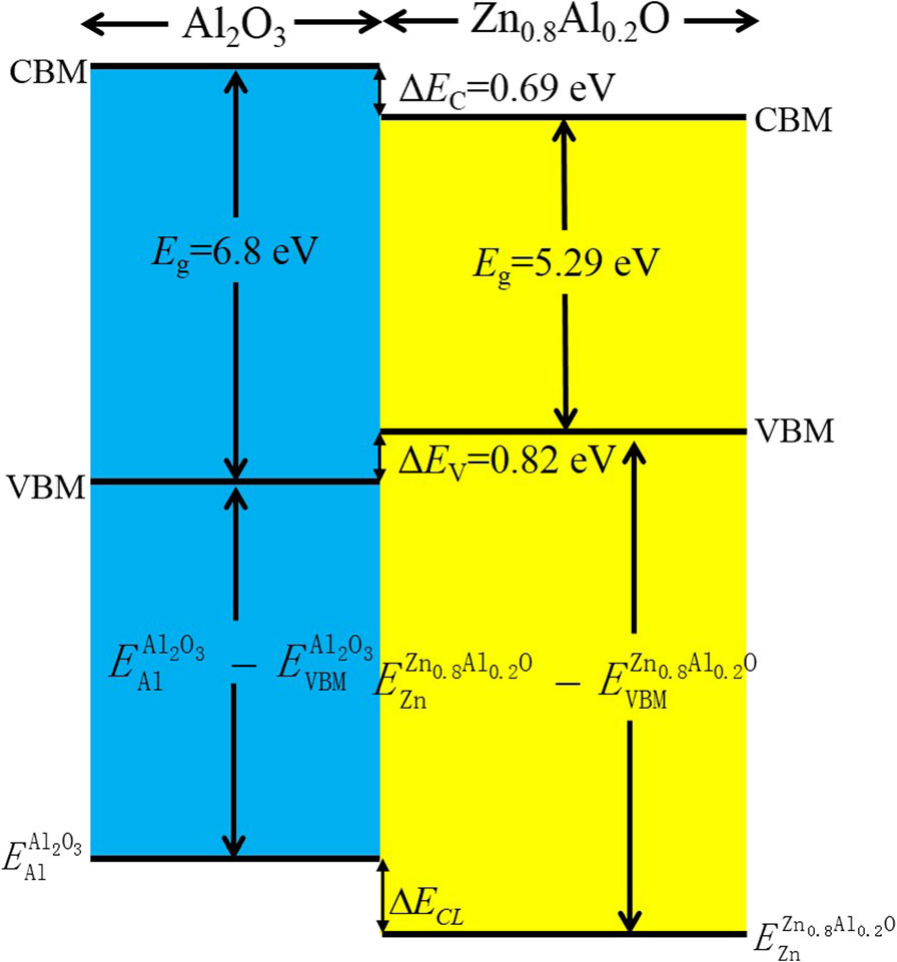
The schematic diagram of band offset at the Al_2_O_3_/Zn_0.8_Al_0.2_O heterojunction interface

To infer the Δ*E*_C_ based on the value of Δ*E*_V_, we need to know the *E*_g_ of the ultrathin Al_2_O_3_ layer, which can be estimated from O 1*s* core-level binding energy spectrum of atomic-layer-deposited Al_2_O_3_ thin films with energy loss structure. The binding energy is calculated from the difference in the total photoelectron energy minus the kinetic energy due to the loss in photoelectron energy by inelastic collision processes within the sample. The minimum inelastic loss is equal to the band gap energy, and the most cited value of *E*_g_ is 6.8 eV [41–43]. Together with the Zn_0.8_Al_0.2_O *E*_g_ of 5.29 eV at room temperature, the Δ*E*_C_ can be simply derived by the equation, *ΔE*_C_ = *E*_g_(Al_2_O_3_) − *ΔE*_V_ − *E*_g_(Zn_0.8_Al_0.2_O), where *E*_g_(Al_2_O_3_) and *E*_g_(Zn_0.8_Al_0.2_O) are the band gaps of the Al_2_O_3_ and Zn_0.8_Al_0.2_O thin films, respectively. The Δ*E*_C_ is calculated to be 0.69 ± 0.12 eV, which means that the barrier height for transport of electrons is smaller than that of holes. The band alignment of the Al_2_O_3_/Zn_0.8_Al_0.2_O heterojunction obtained from XPS measurements is shown in Fig. 7. The CBM of Al_2_O_3_ is higher than that of Zn_0.8_Al_0.2_O; however, the VBM of Al_2_O_3_ is lower than that of Zn_0.8_Al_0.2_O. Therefore, a nested type-I band alignment with a ratio Δ*E*_C_/Δ*E*_V_ of about 1:1.2 is obtained.

Usually, the MCP gain under direct current (DC) mode is limited by the space charge density without consideration of ion feedback, and the recharge time constant or dead time (*τ*) is several milliseconds [44]. When operating a MCP as a DC current amplifier, the gain is constant until the output current (*I*_oc_) exceeds about 10% of the strip current through the plate. However, the MCP works in a highly saturated state under a photon-counting mode, and the electron avalanche multiplication is done within several nanoseconds that is a million times faster than *τ* [44–46]. The peak output current in pulsed operation exceeding the *I*_oc_ by several orders of magnitude is observed. Therefore, anode signal charges probably come from the tunneling electrons in the Al_2_O_3_/Zn_*x*_Al_1-*x*_O heterojunction of the inner wall of the MCP. For photon-counting mode, both Δ*E*_V_ and Δ*E*_C_ should be sufficiently large, which can prevent the thermal excitation of electrons generated from the SEE layer into the electron multiplier system that probably produces high electronics dark noise and result in a reduced signal to noise ratio. The present result has no effects on the MCP operating under DC mode which is determined by space charge saturation, but has negative effects on the photon-counting mode which needs a type-II heterojunction to improve tunneling probability for excellent performance. The relationship between the Al_2_O_3_/Zn_*x*_Al_1-*x*_O heterojunction and charge replenishment mechanism under photon-counting mode needs further study. Therefore, the band alignment of the Al_2_O_3_/Zn_*x*_Al_1-*x*_O heterojunction should be constructed and adjusted by appropriately changing the ratio of Al and Zn elements under the premise of meeting the requirements of the electron multiplier.

## Conclusions

The structure and optical band gaps of Zn_*x*_Al_1-*x*_O (0.2 ≤ *x* ≤ 1) films deposited by atomic layer deposition are investigated. And the band offset measurements of the Al_2_O_3_/Zn_0.8_Al_0.2_O heterojunction have been determined by XPS with zero charging method. The results show that X-ray irradiation time is one of the most important parameters to determine the band offsets. The layer thickness dependence in peak positions is mainly derived from the differential charging effects, and true peak positions are obtained by extrapolating the measured binding energies to zero oxide thickness and ideally to zero charge. The Δ*E*_V_ value is obtained to be 0.82 ± 0.12 eV, and the corresponding Δ*E*_C_ is calculated to be 0.69 ± 0.12 eV. Therefore, a nested type-I band alignment is obtained. Understanding of the band alignment parameters of the Al_2_O_3_/Zn_0.8_Al_0.2_O interface will facilitate the knowledge of their carrier transport mechanism and design of corresponding hybrid devices, especially in the research process of electron multipliers.

## Abbreviations

Al_2_O_3_: Aluminum oxide
ALD: Atomic layer deposition
CBM: Conduction band minimum
CLs: Core levels
DC: Direct current
DEZ: Diethylzinc
*E*_g_: Band gap
*I*_oc_: Output current
MCP: Microchannel plate
MgO: Magnesium oxide
SEE: Secondary electron emission
TMA: Trimethylaluminum
VBM: Valence band maximum
XPS: X-ray photoelectron spectroscopy
XRD: X-ray diffractometer
Zn_*x*_Al_1-*x*_O: Zinc aluminum oxide
Δ*E*_C_: Conduction band offset
Δ*E*_V_: Valence band offset
